# Chinese Dietary Indices and Glioma: New Insights of a Case–Control Study in the Chinese Population

**DOI:** 10.3390/nu15163602

**Published:** 2023-08-17

**Authors:** Weichunbai Zhang, Yongqi He, Feng Chen, Ce Wang, Xun Kang, Yue Peng, Wenbin Li

**Affiliations:** Department of Neuro-Oncology, Cancer Center, Beijing Tiantan Hospital, Capital Medical University, Beijing 100070, China; zwchunbai@163.com (W.Z.); 122021010536@mail.ccmu.edu.cn (Y.H.); chenfeng@bjtth.org (F.C.); 13146851017@163.com (C.W.); kangxuntiantan@163.com (X.K.); pengyue227@163.com (Y.P.)

**Keywords:** Chinese dietary index, glioma, case–control study, dose–response relationship

## Abstract

Identifying modifiable factors in primary prevention strategies is a typical goal of glioma epidemiology. Among many glioma risk factors, diet was always considered as one. Most of the relevant studies thus far were concentrated on the West. It was crucial to investigate the connection between the Chinese diet and gliomas given the stark variations between western and eastern diets. A food frequency questionnaire including 114 items was used to investigate the food intake of the study subjects. The Chinese Dietary Quality Index (CDQI), the Chinese Dietary Balance Index (CDBI), the Dietary Antioxidant Index (DAI), the Dietary Inflammation Index (DII), and the Chinese Healthy Eating Index (CHEI) were calculated based on the data provided by the food frequency questionnaire to evaluate dietary quality, dietary balance, dietary antioxidants, dietary inflammation and adherence to the Chinese dietary guidelines in 506 glioma patients and 506 controls, respectively. After adjusting covariates, CHEI (OR = 0.90, 95% CI: 0.88–0.93) and DAI (OR = 0.61, 95% CI: 0.54–0.70) were correlated to a reduced glioma risk, and CDBI-based undernutrition (OR = 1.08, 95% CI: 1.06–1.12) and overnutrition (OR = 1.14, 95% CI: 1.09–1.20) and DII (OR = 2.20, 95% CI: 1.81–2.68) were correlated to an elevated glioma risk. Moreover, restrictive cubic spline analysis showed that there were significant nonlinear dose–response relationships between CHEI, CDBI, DAI, DII, and glioma. Therefore, adhering to the Chinese dietary guidelines was connected with a lower glioma risk, and undernutrition and overnutrition in the Chinese diet were associated with an increased risk of glioma.

## 1. Introduction

The majority of initial malignant tumors of central nervous system are gliomas, which are caused by cancerous changes in the brain cells, accounting for about 80.8% cases [1]. Although glioma had an extremely low incidence (5.7/100,000), its prognosis was extremely poor compared with other cancers, and even under active treatment, the median survival of glioblastoma, which had the highest degree of malignancy, is only 8 months, and the five-year survival rate did not exceed 7% [1]. In the last ten years, the treatment of glioma has made great progress, but considering its severe disease burden, exploring the influencing factors in the primary prevention strategy of glioma was still one of the main objectives of epidemiological studies on brain tumors [2].

To date, exposure to pesticides [3], allergic diseases [4], and certain rare genetic mutations [5] have been link to the incidence of glioma, but exposure to only specific doses of ionizing radiation have been recognized as a cause of the disease [6]. As one of the most closely related life behaviors to health, diet, and cancer has always attracted much attention [7,8,9]. But, in contrast, the study on gliomas was inadequate. Some early studies have found that antioxidant-rich foods such as vegetables (especially green and orange) [10], fruits [11], and nuts [12] were link to a lower risk of glioma. Drinking a moderate amount of tea or coffee every day had a similar effect [13,14]. Excessive consumption of processed meats and refined grains may be risk factors for gliomas [15,16]. The corresponding results have also been validated in meta-analyses [17]. However, inconsistent results were not found in prospective cohort studies [18,19,20]

The reason for this contradiction may be that the comprehensive effect of diet was not considered. There were complex interactions and cumulative relationships between foods, such as when the consumption of some foods increased while the consumption of others declined [21,22,23]. Therefore, measuring a combination of integrated diets may be more consistent with dietary realities than assessing a single food group or nutrient. Although these studies investigated the effects of certain food groups on gliomas, few studies evaluated the connections of diet as a whole with gliomas. Currently, adhering to the Mediterranean diet was found to significantly reduce glioma risk in Iran (odds ratio (OR) = 0.36, 95% confidence interval (95% CI): 0.16–0.78) [24]. But adherence to the American dietary guidelines was not found to have a significant effect on gliomas in cohort studies [25]. Moreover, the assessment of dietary patterns in these studies was based on the local population, which may not be applicable to East Asia and the Western Pacific region.

Therefore, in order to overcome the limitations of single food groups and avoid food interactions, we used the dietary indices to comprehensively assess the overall diet. Based on the Chinese dietary indices constructed from previous studies, we used the Chinese Dietary Quality Index (CDQI) [26] to assess the dietary quality, the Chinese Dietary Balance Index (CDBI) [27] to assess the dietary balance, and the Chinese Healthy Eating Index (CHEI) [28] to assess the extent to which individuals adhered to the dietary guidelines for Chinese residents. Considering that the underlying pathogenesis of glioma was closely related to oxidative stress and inflammation, we also assessed the antioxidant and proinflammatory capacity from foods using the Dietary Antioxidant Index (DAI) [29] and the Dietary Inflammation Index (DII) [30]. Based on the above five dietary indices, this study aimed to explore the association between Chinese dietary indices and glioma in many aspects, and further intuitively quantify the relationship between these dietary indices and glioma risks through dose–response relationships, so as to supplement the epidemiological evidence of Chinese dietary indices for primary prevention of glioma.

## 2. Materials and Methods

### 2.1. Study Population

This was a case–control study based on the Chinese population conducted at Beijing Tiantan Hospital. Neuro-oncologists and pathologists collaboratively defined glioma patients in the case groups using the 2021 Diagnostic Criteria [31]. Based on previous studies, we assumed that about 75% of the Chinese population did not adhere well to the Chinese dietary guidelines [28]. Due to the lack of epidemiological studies on the relationship between Chinese dietary indices and glioma, we referred to the effect size between a healthy diet index and glioma and concluded that a healthy diet was associated with a 74% reduction in the risk of glioma [32]. When the power was 80% and the type I error was 0.05, we calculated a minimum sample size of 103 cases and 103 controls. All participants included in the study were required to be at least 18 years old. Individuals with abnormal calorie consumption (less than 400 or greater than 5000 kilocalorie/day), women during gestation period, significant dietary changes (e.g., weight loss) before the survey, any previous cancer (except glioma), diseases such as digestion, endocrine, and nervous systems, unable to complete the survey due to cognitive impairment, and consuming hormones and other diet-disrupting medications were not involved in this study. The control group consisted of healthy people from communities and matched glioma patients 1:1 by sex and age (±5 years) under the conditions that met the above criteria. Among them, 15 subjects in the case group refused to participate after understanding the purpose of the study, and the response rate was 97.2%. In the control group, 41 subjects refused to participate after knowing the purpose of the study, and the response rate was 92.7%. On this basis, eleven subjects (six subjects in the case group and five subjects in the control group) with severe questionnaire information missing, four subjects with cognitive impairment unable to complete the survey (four subjects in the case group), two subjects with absent pathological information (two subjects in the case group), and nine subjects with mismatch (nine subjects in the control group) were excluded. At last, 506 pairings in all were taken into consideration for the statistical study (Figure S1). Beijing Tiantan Hospital’s Institutional Review Board approved the study (No. KY2022-203-02).

### 2.2. Dietary Assessment

The Food Frequency Questionnaire (FFQ) served as the primary method for evaluating dietary patterns in this study. We adapted the questionnaire from the FFQ utilized in the China National Nutrition and Health Survey [33]. With that in mind, we added and removed several foods from the papers on food and glioma to make them more relevant to the purpose of the study, and the final questionnaire included 114 items (Table S1). To ensure the quality of dietary surveys, we also re-verified the revised FFQ. The correlation coefficients of the food group and nutrients were between 0.380 and 0.847 [34], suggesting that the FFQ provided a reasonable and effective measurement method for assessing long-term dietary intake. The vast majority of cases had dietary surveys during the period before chemotherapy after pathological diagnosis. To maintain consistency, trained investigators employed the same face-to-face approach for conducting dietary surveys with both healthy individuals and glioma patients. During the surveys, investigators utilized visual aids, such as images depicting various meal types and quantities, to enhance the accuracy of data collection. Subjects required to complete the food intake frequency and average intake each time in the FFQ in the past year. Each food’s average daily intake (g/d or ml/d) was computed according to the frequency and average intake of each food for statistical analysis.

Nutrient intakes involved in this study were calculated from daily food intakes and the China Food Composition Table [35]. This was currently the most authoritative and comprehensive food composition reference in China, which provided the amount of nutrients in each food. By multiplying the daily consumption of different foods by their respective nutrient values and summing these amounts, we derived the total nutrient intake for each subject. Energy intake was similar.

### 2.3. Evaluation of Dietary Indices

Based on previous studies, existing dietary assessment methods [23], and health-related nutritional knowledge, we assessed the Chinese Dietary Quality Index (CDQI) [26], the Chinese Dietary Balance Index (CDBI) [27], the Dietary Antioxidant Index (DAI) [29], the Dietary Inflammation Index (DII) [30], and the Chinese Healthy Eating Index (CHEI) [28]. Among them, CHEI represented the evaluation of the degree of consistency between individual diets and the Chinese dietary guidelines. DAI represented an indicator for evaluating the overall antioxidant capacity of the diet. DII represented an indicator that evaluated the overall pro-inflammatory ability of the diet. CDQI represented the evaluation of individual dietary quality based on nutrient intake, while CDBI represented the evaluation of individual dietary balance based on food group intake. These two indicators were specifically divided into low bound score (LBS), high bound score (HBS), and dietary quality distance (DQD). LBS represented the overall situation of insufficient dietary intake, i.e., undernutrition, while HBS represented the overall situation of excessive dietary intake, i.e., overnutrition, DQD represented the overall situation of imbalanced dietary intake (taking into account both insufficient and excessive intake), and further away the score was from 0, the more severe the situation of excessive or insufficient nutrition was. Supplementary Material A provides specific evaluation methods.

### 2.4. Other Variables

To assess potential confounders, we also collected information from questionnaires on other variables, including age (continuous variable), sex (categorical variable), household income (categorical variable), occupation (categorical variable), education level (categorical variable), history of allergies (categorical variable), history of head trauma (categorical variable), family history of cancer (categorical variable), smoking status (categorical variable), high-risk residential area (categorical variable), and physical activity (categorical variable). The histories of the three diseases were judged by the presence or absence of the individual or family. In the last ten years, high-risk residential areas have been classified as regions where people live close to electromagnetic fields and broadcast antennae [36]. Physical activities were evaluated and classified in accordance with an international questionnaire [37].

In addition, field investigators measured the height and weight of the study subjects using standardized procedures and reliable equipment to estimate the body mass index (BMI) by weight (kg) and square of the height (m^2^).

### 2.5. Statistical Analysis

The percentages (%) were reported for counting data. Measurement data with severely skewed distributions were reported using median and quartile, whereas variables with normal distributions were reported using mean and standard deviation. To compare groups of measurement data, the *t*-test and Mann–Whitney U test were used, the former for data with a normal distribution and the latter for data with a skewed distribution. To determine if counting data differed between the glioma and healthy groups, the chi-square test was employed. The link between these dietary indices was assessed using the Spearman correlation coefficient.

We constructed multiple logistic regression to assess the connections between glioma and Chinese dietary indices, as well as calculated ORs and 95% confidence intervals (95% CI). Multiple logistic regression was introduced for the categorical variables (tertiles) and continuous variables of CHEI, CDQI (LBS, HBS, and DQD), CDBI (LBS, HBS, and DQD), DAI, and DII. There was no covariate adjustment made to Model 1. Model 2 was adjusted for household income, physical activity, occupation, education level, history of allergies, family history of cancer, history of head trauma, smoking status, and high-risk residential area (the above were all categorical variable), and age, energy intake, and BMI (these three variables were all continuous variable).

To assess the reliability of the test results, we performed a number of sensitivity studies. The above model was mainly repeated by excluding some people who have different BMI, different sex, different age, lower education, lower income, smoking, and allergy or tumor family history. In addition, considering that the relationship between BMI and diet may indirectly affect glioma, causal mediation analysis was used to explore the mediating effect of BMI between Chinese dietary indices and glioma [38,39]. Each dietary index’s potential dose–response association with the risk of glioma was simulated through the restriction cubic spline (RCS), with each node distributed every 20 percentiles of their distribution for a total of four nodes. Reference values set in the places reported in the articles for CDQI, CDBI, and DII (0 points), and reference values set in the 10th percentile (OR = 1) for CHEI and DAI [40].

SPSS 26.0 and R software 4.1.1 were used to complete all statistical analyses. All statistical tests were two-sided, and statistical significance was determined by *p* < 0.05.

## 3. Results

### 3.1. Characteristics of the Study Population and Dietary Indices

This study included 1012 participants, with 506 cases in the glioma group. The glioma group was composed of 104 astrocytomas, 67 oligodendrogliomas, 237 glioblastomas, 18 diffuse midline gliomas, and 80 others, with an average age of 42.62 ± 13.09 years. The healthy group had an average age of 41.15 ± 12.85 years, and the sex composition of the two groups was highly consistent. The glioma group had a higher BMI (*p* < 0.001), a higher proportion of manual workers (*p* = 0.024), lower education levels (*p* < 0.001), a higher proportion of smoking (*p* = 0.039), and a higher proportion of high physical activity (*p* < 0.001) compared to the healthy group. Additionally, there were differences in household economic income (*p* < 0.001), with a lower proportion of allergy history (*p* < 0.001) and a higher family history of cancer (*p* = 0.001). The other variables showed no differences that were statistically significant (Table 1).

**Figure 1:**
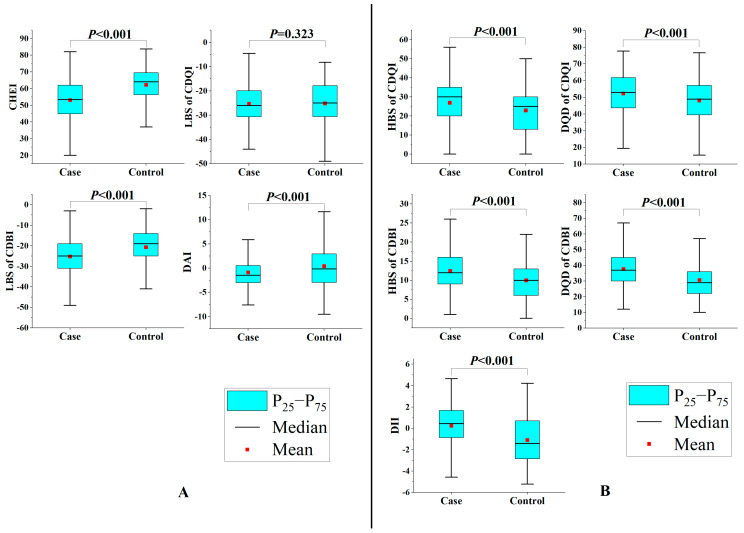
Distribution of Chinese dietary indices between case and control groups. (**A**) Healthier diet; (**B**) less healthy diet. *P*-values were unadjusted and derived from Mann–Whitney U test. Abbreviation: CDQI, Chinese Dietary Quality Index; CDBI, Chinese Dietary Balance Index; CHEI, Chinese Healthy Eating Index; DAI, Dietary Antioxidant Index; DII, Dietary Inflammation Index; DQD, dietary quality distance; HBS, high bound score; LBS, low bound score.

For dietary intake, compared to the average dietary intake of the representative population in China, for the case group, the intakes of grains, vegetables, red meat, and fish were lower, while the intakes of fruits and eggs were higher. The intakes of tubers, legumes, poultry, animal offal, and nuts were basically the same. For the control group, the intakes of tubers, legumes, poultry, fish, and nuts were basically the same, while the intakes of grains and red meat were lower, while the intakes of vegetables, fruits, animal offal, and eggs were higher (Table S2). For dietary index, as shown in Figure 1, when cases were compared, CHEI and LBS of CDBI and DAI were higher in the control group (*p* < 0.001), but HBS of CDQI, DQD of CDQI, HBS of CDBI, DQD of CDBI, and DII were significantly lower (*p* < 0.001). LBS of CDQI did not differ significantly (*p* = 0.323). In addition, there was a certain correlation between these dietary indices. Correlation coefficients ranged from −0.906 to 0.793 (Table S3).

### 3.2. Chinese Dietary Indices and Glioma

The findings of the correlation between Chinese dietary indices and gliomas are presented in Table 2. After adjusting covariates (Model 2), the continuous variable analysis of dietary index showed that each one-point rise in CHEI had an association with a 10% reduction in the glioma risk (OR = 0.90, 95% CI: 0.88–0.93), each one-point rise in HBS of CDQI had an association with a 3% increment in the glioma risk (OR = 1.03, 95% CI: 1.01–1.05), each one-point rise in DQD of CDQI had an association with a 3% increment in the glioma risk (OR = 1.03, 95% CI: 1.01–1.05), each one-point decrease in LBS of CDBI had an association with an 8% increment in the glioma risk (OR = 1.08, 95% CI: 1.06–1.12), each one-point rise in HBS of CDBI had an association with a 14% increment in the glioma risk (OR = 1.14, 95% CI: 1.09–1.20), each one-point rise in DQD of CDBI had an association with an 8% increment in the glioma risk (OR = 1.08, 95% CI: 1.06–1.11), each one-point rise in DAI had an association with a 39% reduction in glioma risk (OR = 0.61, 95% CI: 0.54–0.70), and each one-point rise in DII had an association with a 120% increased risk of glioma (OR = 2.20, 95% CI: 1.81–2.68). However, the association between LBS of CDQI and glioma was not significant. In addition, the results of the categorical variable for the dietary index were similar to those described above.

### 3.3. Chinese Dietary Indices and Gliomas of Different Pathological Classifications

The outcomes of pathological subtypes are shown in Table 3. For astrocytoma, higher CHEI (OR = 0.89, 95% CI: 0.83–0.96) and DAI (OR = 0.01, 95% CI: 0.001–0.60) were linked to a reduced risk, lower LBS of CDBI (OR = 1.16, 95% CI: 1.06–1.27) was linked to an elevated risk, and higher HBS of CDBI (OR = 1.19, 95% CI: 1.05–1.34), DQD of CDBI (OR = 1.17, 95% CI: 1.07–1.29), and DII (OR = 5.49, 95% CI: 1.92–15.69) were linked to an elevated risk. For glioblastoma, higher CHEI (OR = 0.83, 95% CI: 0.77–0.90) and DAI (OR = 0.71, 95% CI: 0.59–0.87) were linked to a reduced risk, lower LBS of CDBI (OR = 1.14, 95% CI: 1.06–1.22) was linked to an elevated risk, and higher HBS of CDBI (OR = 1.12, 95% CI: 1.04–1.22), DQD of CDBI (OR = 1.10, 95% CI: 1.05–1.15), and DAI (OR = 2.21, 95% CI: 1.52–3.20) were linked to an elevated risk. However, the correlation index of CDQI was not significantly associated with different pathological classifications of glioma. The tiny sample size of oligodendroglioma precluded further study (Table 3).

### 3.4. Chinese Dietary Indices and Glioma of Different Pathological Grades

The analysis presented in Table 4 revealed consistent findings concerning the association between pathological grades and dietary indices (Table 4). For low-grade gliomas, higher CHEI (OR = 0.91, 95% CI: 0.86–0.96) and DAI (OR = 0.27, 95% CI: 0.12–0.61) were linked to a reduced risk, lower LBS of CDBI (OR = 1.12, 95% CI: 1.04–1.20) was linked to an elevated risk, and higher HBS of CDBI (OR = 1.29, 95% CI: 1.08–1.54), DQD of CDBI (OR = 1.13, 95% CI: 1.05–1.22), and DII (OR = 3.21, 95% CI: 1.58–6.52) were linked to an elevated risk. For high-grade gliomas, higher CHEI (OR = 0.87, 95% CI: 0.83–0.91) and DAI (OR = 0.69, 95% CI: 0.59–0.81) were linked to a reduced risk, lower LBS of CDBI (OR = 1.10, 95% CI: 1.05–1.15) was linked to an elevated risk, and higher DQD of CDQI (OR = 1.03, 95% CI: 1.01–1.06), HBS of CDBI (OR = 1.14, 95% CI: 1.07–1.22), DQD of CDBI (OR = 1.09, 95% CI: 1.05–1.13), and DII (OR = 2.20, 95% CI: 1.67–2.89) were linked to an elevated risk. However, the other indices of CDQI were not significantly associated with pathological grades.

### 3.5. Sensitivity Analysis

Sensitivity analysis revealed that the results of CHEI, LBS of CDQI, CDBI, DAI, and DII corresponded with the results of the total population after excluding specific participants. However, the results of HBS of CDQI and DQD of CDQI were not significant in ≤40-year-olds (Table S4).

Causal mediating analysis revealed that BMI played little mediating role in the effects of these dietary indices on glioma (Table S5).

### 3.6. Dose–Response Relationship

**Figure 2:**
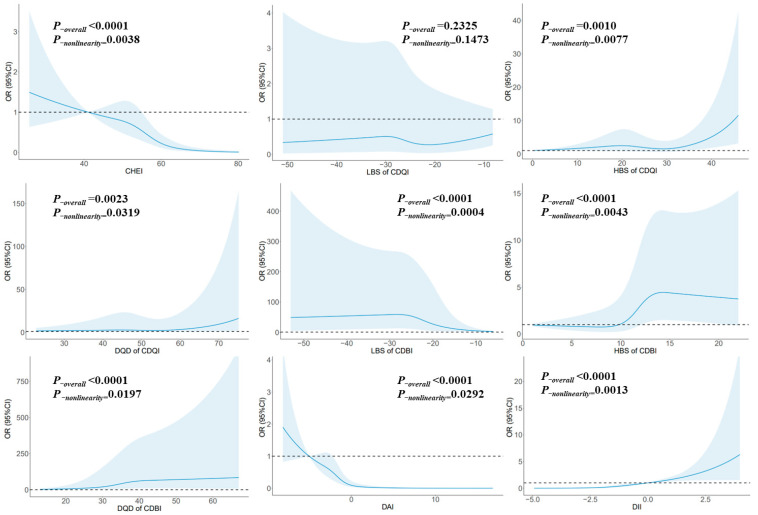
The RCS for the associations between Chinese dietary indices and glioma. The lines represent adjusted odds ratios based on RCSs for the intake in the regression model. Knots were placed at the 20th, 40th, 60th, and 80th percentiles of Chinese dietary indices, and reference values set in the places reported in the articles for CDQI, CDBI, and DII (0 points), and reference values set in the 10th percentile (OR = 1) for CHEI and DAI. The adjusted factors were the same as in Model 2. The dotted line represented OR=1, and the solid blue lines represented the effect values.

Figure 2 illustrates the relationship between glioma and each dietary index using RCS. Notably, CHEI exhibited a nonlinear dose–response relationship. Risk reduction was observed when the score surpassed 55.0, signifying a considerable decline in risk with higher scores (*P_-nonlinearity_* = 0.0038). Conversely, HBS of CDQI displayed a nonlinear dose–response relationship, with a notable increase in risk once the score exceeded 35.0 (*P_-nonlinearity_* = 0.0077). Furthermore, DQD of CDQI indicated a nonlinear dose–response relationship, wherein risk escalated significantly when the score surpassed 64.28 (*P_-nonlinearity_* = 0.0319). Conversely, LBS of CDBI exhibited a nonlinear dose–response relationship. Risk reduction was evident when the score exceeded −28.0, pointing to a substantial decrease in risk with higher scores (*P_-nonlinearity_* = 0.0004). Moreover, HBS of CDBI demonstrated a nonlinear dose–response relationship. Risk significantly increased once the score surpassed 13.0, and after reaching a score of 15.0, the risk remained relatively stable (*P_-nonlinearity_* = 0.0043). Similarly, DQD of CDBI displayed a nonlinear dose–response relationship, with risk consistently increasing with higher scores (*P_-nonlinearity_* = 0.0197). Additionally, DAI showcased a nonlinear dose–response relationship. Risk reduction was notable when the score exceeded −2.23, and after reaching a score of 3.3, the risk remained relatively stable (*P-_nonlinearity_* = 0.0292). Finally, DII revealed a nonlinear dose–response relationship, with the risk increasing significantly as scores escalated (*P_-nonlinearity_* = 0.0013).

## 4. Discussion

Our research fully explored the relationship between Chinese dietary indices and glioma from a new perspective. Among them, CHEI, CDBI, and DAI had an association with a reduced risk of glioma, while HBS of CDBI, DQD of CDBI, and DII had an association with an increased risk of glioma. Significant nonlinear connections between these dietary indices and glioma were further supported by the dose–response relationship as described by the RCS. In addition, the sensitivity analysis suggested that the results of HBS of CDQI and DQD of CDQI were not robust.

CHEI is a dietary index specified based on food entries and recommended intakes in the Chinese Dietary Guidelines, which can well measure compliance with the Chinese Dietary Guidelines. So far, the effects of CHEI on cancer have been documented in a number of research. By comparing the CHEI of 720 liver cancer patients and healthy individuals, Chen et al. discovered that the probability of primary liver cancer dropped by 57% (OR = 0.43, 95% CI: 0.38–0.50) with each five-point rise in score [41]. Luo et al. also found that in the Guangdong Cohort Study, 887 patients with liver cancer were followed for 797 days, and higher CHEI scores were substantially linked with mortality from all causes (hazard ratio (HR) = 0.75, 95% CI: 0.58–0.98) and mortality from liver cancer (HR = 0.74, 95% CI: 0.56–0.98) [42]. In addition, Maitiniyazi’s study also found that adhering to Chinese dietary guidelines can help relieve mental symptoms and discomfort in breast cancer patients [43]. However, there were no reports of neuro-oncology. In this study, it was found for the first time that adherence to Chinese dietary guidelines had an association with a reduced glioma risk (OR = 0.90, 95% CI: 0.88–0.93), and had a greater effect on high-grade gliomas (OR = 0.87, 95% CI: 0.83–0.91), such as glioblastoma (OR = 0.83, 95% CI: 0.77–0.90), and the RCS also showed a nonlinear dose–response relationship. When the CHEI score exceeded 55.0, the glioma risk reduced significantly with the elevated score (*P_-nonlinearity_* = 0.0038). In past studies, Sadeghhi et al. reported a significant association between the higher Healthy Eating Index (HEI, an index of adherence to the American dietary guidelines) and gliomas in a population study in West Asia (OR = 0.26, 95% CI: 0.12–0.56) [32]. Not only did the formulation of CHEI refer to HEI, but the two dietary guidelines shared similarities in terms of composition. They both encouraged eating more whole grains, vegetables, seafood, dairy, legumes, and fruits while limiting sodium and added sugars [44]. Therefore, the results of the two studies were mutually corroborated. Moreover, independent studies involving the above food groups and gliomas have also reported that they were consistent with the direction of dietary guidelines [10,11,12]. However, a similar correlation was not found in the cohort study. Kuan et al. combined analysis of three sizable population studies from the US and the UK and found no significant association between alternative healthy dietary indices and gliomas (relative risk (RR) = 1.06, 95% CI: 0.91–1.23), which was thought to be related to dietary differences in different populations [25]. In addition, other dietary patterns that were similar in composition to the Chinese Dietary Guidelines, such as the DASH diet, etc., have also been discovered to be linked to a lower incidence of glioma [24,45].

Unlike dietary patterns, both CDQI and CDBI were indices that assess dietary quality and balance. Although these indices were based on the dietary quality indices of western countries [46], considering the situation of undernutrition and overnutrition in developing countries at the same time, developing countries need dietary quality indicators that are tailored to their requirements and can be adapted to different nutritional imbalances. Although studies of dietary quality and cancer have found that better dietary quality had an association with a lower cancer mortality (RR = 0.91, 95% CI: 0.89–0.93) [47] and a lower breast cancer risk (OR = 0.21, 95% CI: 0.07–0.62) [48], but studies of dietary quality and gliomas were rare. Shayanfar et al. introduced the Index of Nutritional Quality to analyze the connection between Iranian dietary status and glioma, but this indicator can only evaluate a single nutrient and cannot consider the overall impact of the diet [49]. Our study has compensated for this. For CDQI, the results were significant for HBS of CDQI (OR = 1.03, 95% CI: 1.01–1.05) and DQD of CDQI (OR = 1.03, 95% CI: 1.01–1.05) alone, but not significant in any pathological subgroups. For CDBI, LBS, HBS, and DQD were significant in both the population and subgroups, suggesting that undernutrition and overnutrition had an association with an elevated glioma risk. It was speculated that it was related to oxidative stress caused by undernutrition and overnutrition, which may disrupt oxidative homeostasis, mediate elevated reactive oxygen species levels, and stimulate cancer initiation by leading to DNA mutations, damage, and cancer-promoting signals [50]. In contrast, it seemed that overnutrition had a greater impact on glioma than undernutrition, which was related to obesity and increased intake of dietary chemical carcinogens caused by overnutrition [51,52,53]. In addition, the correlation results suggested that a balanced diet had a higher antioxidant capacity and anti-inflammatory capacity, which may also be responsible for the reduction in glioma risk in a high-quality diet. Although the results of CDQI and CDBI were inconsistent, they were not contradictory. They were all used to measure the dietary quality in the Chinese Population, but there were large differences in the composition of indicators. The components of CDQI focused on macronutrients, energy, and two common minerals (sodium and calcium), while the components of CDBI were based on the intake of food groups. Therefore, this study seemed to suggest that imbalances in dietary intake caused a stronger effect on glioma than imbalances in nutrient intake. On the one hand, although the assessment of CDQI covered the main nutrients, some nutrients with potential glioma prevention effects have not been assessed, such as various vitamins, unsaturated fatty acids, and phytochemicals [54,55]. CDBI based on food groups can compensate for this deficiency in the course of dietary assessment. On the other hand, CDBI considered the amount of drinking water, which was not included in most dietary indices [44,56,57]. Because water intake was strongly associated with cancer [58,59], assessing water intake in dietary indicators may be more rigorous.

For a long time, the potential antioxidant effect of diets on health cannot be ignored. Because the consumption of foods or nutrients that have antioxidant properties was closely associated with one another, it was difficult to accurately assess independent effects. Therefore, the DAI was introduced to assess the antioxidant capacity in the whole diet [60]. Moreover, cancer, particularly cancer of the digestive system, has been discovered to be highly correlated with DAI. According to Vahid et al.’s observational study, a higher DAI was linked to lower chances of colorectal cancer (OR = 0.91, 95% CI: 0.85–0.98) [61] and gastric cancer (OR = 0.64, 95% CI: 0.43–0.95) [29]. Similar results were obtained in prospective studies about colorectal cancer in Singapore (HR = 0.80, 95% CI: 0.66–0.98), particularly in women (HR = 0.66, 95% CI: 0.48–0.90) [62]. Furthermore, DAI was also closely related to breast cancer [63], cervical cancer [64], and lung cancer [60]. Similar to our findings (OR = 0.61, 95% CI: 0.54–0.70), Heydari et al. initially showed that DAI was contrarily linked with glioma risk (OR = 0.13, 95% CI: 0.05–0.35) [65], and we also found that the impact of DAI on low-grade glioma was more significant (OR = 0.27, 95% CI: 0.12–0.61). However, our assessment method of DAI was different from that of Heydari whose study focused more on vitamins, such as B vitamins, and did not seem to consider minerals. The above DAI in our study also considered some minerals with antioxidant effects, such as manganese, selenium, and zinc. This was in line with how a prior study had conducted its examination on DAI and glioma prognosis [66]. Il’yasova et al. followed up on 814 glioblastoma patients and found a substantial link between improved survival and increased DAI (HR = 0.58, 95% CI: 0.46–0.74) [66]. Although the possible mechanisms of dietary antioxidation to prevent glioma were very limited, there were still several suggestions for these mechanisms. On the one hand, oxidative stress was closely associated with the formation or growth of cancer [67,68], and diet might decrease the level of oxidative stress markers and improve plasma antioxidant capacity [69]. On the other hand, antioxidants in the diet can inhibit the production of potential carcinogens, such as N-nitroso compounds [70]. In addition, it may also be related to dietary polyphenols with antioxidant activity, such as resveratrol [54,71], apocynum venetum polyphenols [72], etc. These substances had neuroprotective effects on the brain [73], and played a similar role in glioma, including inducing glioma cell apoptosis [74], reducing its invasiveness [74], inhibiting its proliferation through NF-κB pathway [72], and improving the efficacy of radiotherapy and temozolomide [71]. In previous studies, we also found that higher dietary resveratrol intake was associated with a reduced risk of glioma, and there was a significant nonlinear dose–response relationship between them [75], which was very similar to the relationship between resveratrol and other cancers [76]. More importantly, the research on a Mediterranean diet rich in polyphenols has also found a close relationship between adhering to a Mediterranean diet and reducing the risk of glioma [24]. Considering the antioxidant protective effects of polyphenols and other phytochemicals in clearing free radicals of DNA damage and regulating DNA repair mechanisms, we may not be able to ignore the impact of these phytochemicals on glioma.

The DII was developed by American researchers in a cancer prevention and control project. It was a dietary tool derived from the literature to evaluate the ability of individuals’ diets to cause body inflammation [30]. In recent years, several cancer-related studies have been reported. Based on 868 participants, Sasanfar et al. found that a higher DII was linked to a higher chance of developing breast cancer (OR = 1.56, 95% CI: 1.04–2.35), particularly in premenopausal women (OR = 1.92, 95% CI: 1.14–3.25) [77]. Lozano-Lorca et al.’s multicentric study in the Spanish population found that highly inflammatory diets might cause prostate cancer (OR = 1.30, 95% CI: 1.03–1.65) [78]. A meta-analysis that included nine studies showed that colorectal cancer risk increased by 7% for each one-point rise in the DII [79]. Although this dietary index was widely used in cancer prevention research, it was rare for gliomas. Aminianfar et al. found that participants with the highest score of DII had a 1.76-fold increase in the glioma risk (OR = 2.76, 95% CI: 1.15–6.60) by analyzing the diets of 128 newly diagnosed glioma cases and 256 control groups [80]. Our study found similar results in the Chinese population (OR = 2.20, 95% CI: 1.81–2.68), and also obtained the same conclusion in different grades of gliomas. In addition, the dose–response relationship suggested that dietary anti-inflammatory effects (the negative range of the dietary inflammatory index) were protective against gliomas, while dietary pro-inflammatory effects (the positive range of the dietary inflammatory index) might be a hazard factor for gliomas. Since chronic inflammation may be one of the potential mechanisms for the occurrence and development of cancer [81], this was no exception for gliomas. Inflammatory cells and mediators, such as cytokines and chemokines, facilitate the proliferation, angiogenesis, and invasion of gliomas [82,83,84]. Epidemiological studies have also shown that using non-steroidal anti-inflammatory medications was associated with a 33% reduced glioma risk (OR = 0.67, 95% CI: 0.47–0.96) [85]. Since diets with a higher inflammatory index also promoted the development of chronic inflammation in the body [30], this may increase the potential risk of glioma.

The limitation of our study was study type. The study was a case–control design and could not determine a causal relationship between Chinese dietary indices and glioma. However, considering the extremely low incidence of glioma, case–control studies were still one of the main epidemiological methods for the study of the etiology of glioma. We also attempted to minimize the influence of selection bias and recall bias by selecting new cases and conducting dietary surveys with the help of dietary pictures. Second, we can only assess the antioxidant capacity and inflammatory effects of the diet from the perspective of dietary exposure and have not been able to further assess their levels in the body by examining biological samples. In addition, due to the limitation of the items in the FFQ, some inflammation-related foods could not be evaluated, and there were still other potential confounding factors that have not been considered, such as genetic factors, exposure to ionizing radiation such as CT, etc. However, based on existing papers and the actual situation of this study, these factors did not cause serious confounding bias in this study. However, this study still had several advantages. Firstly, our investigation was the first to evaluate the relationship between Chinese dietary indices and glioma using a dietary index. Considering the differences between eastern and western eating habits, the results of this study were representative. Secondly, this study also revealed the association between glioma and diet from the perspective of dietary balance for the first time. This result suggested that nutritional imbalance may be a risk factor for glioma, especially overnutrition. Moreover, we provided results for different pathotypes and grades, as well as dose–response relationships, which have rarely been reported in previous studies.

## 5. Conclusions

In summary, we observed that adherence to the Chinese dietary guidelines had an association with a reduced glioma risk, while undernutrition or overnutrition due to dietary imbalance had an association with an elevated glioma risk, which could be connected to the antioxidant and pro-inflammatory capacity of the diet. According to the Chinese dietary indices, maintaining a balanced and high-quality diet could potentially contribute to the prevention of glioma occurrence and progression.

## Figures and Tables

**Table 1:** Basic characteristics of the case group and control group (*n* = 1012).

	Case (*n* = 506)	Control (*n* = 506)	*p*-Value ^a^
Age (years)	42.62 ± 13.09	41.15 ± 12.85	0.072
Sex, *n* (%)			1.000
Male	284 (56.1)	284 (56.1)	
Female	222 (43.9)	222 (43.9)	
BMI (kg/m^2^)	24.03 ± 3.25	23.05 ± 3.27	<0.001
High-risk residential area, *n* (%)			0.534
Yes	108 (21.3)	100 (19.8)	
No	398 (78.7)	406 (80.2)	
Occupation, *n* (%)			0.024
Manual workers	134 (26.5)	103 (20.4)	
Mental workers	265 (52.4)	306 (60.5)	
Others	107 (21.1)	97 (19.1)	
Education level, *n* (%)			<0.001
Primary school and below	35 (6.9)	13 (2.6)	
Middle school	210 (41.5)	127 (25.1)	
University and above	261 (51.6)	366 (72.3)	
Household income, *n* (%)			<0.001
<3000 CNY/month	49 (9.7)	92 (18.2)	
3000–10,000 CNY/month	384 (75.9)	249 (49.2)	
>10,000 CNY/month	73 (14.4)	165 (32.6)	
Smoking status, *n* (%)			0.039
Never smoked	354 (70.0)	381 (75.3)	
Former smoker	65 (12.8)	41 (8.1)	
Current smoker	87 (17.2)	84 (16.6)	
History of allergies, *n* (%)			<0.001
Yes	39 (7.7)	74 (14.6)	
No	467 (92.3)	432 (85.4)	
History of head trauma, *n* (%)			0.474
Yes	57 (11.3)	50 (9.9)	
No	449 (88.7)	456 (90.1)	
Family history of cancer, *n* (%)			0.001
Yes	152 (30.0)	107 (21.1)	
No	354 (70.0)	399 (78.9)	
Physical activity, *n* (%)			<0.001
Low	69 (13.6)	232 (45.8)	
Moderate	209 (41.3)	184 (36.4)	
High	228 (45.1)	90 (17.8)	

**^a^** *p*-values were derived from Student’s *t*-tests for continuous variables according to the data distribution and the chi-square test for the classified variables.

**Table 2:** Adjusted Ors and 95% CI for the association between the categorical variable and continuous variables of the Chinese dietary indices and glioma.

Dietary Index	T1	T2	T3	Continuous ^c^
CHEI	<53.48	53.48–64.30	>64.30	
Case/Control	254/84	160/177	92/245	
Model 1 ^a^	1	**0.30 (0.21–0.43)**	**0.12 (0.08–0.18)**	**0.93 (0.91–0.94)**
Model 2 ^b^	1	**0.31 (0.17–0.57)**	**0.06 (0.03–0.13)**	**0.90 (0.88–0.93)**
DAI	<−2.43	−2.43–0.60	>0.60	
Case/Control	186/152	195/142	125/212	
Model 1 ^a^	1	1.12 (0.81–1.55)	**0.50 (0.37–0.69)**	**0.94 (0.91–0.97)**
Model 2 ^b^	1	**0.51 (0.27–0.97)**	**0.08 (0.03–0.18)**	**0.61 (0.54–0.70)**
LBS of CDQI	<−22.08	−22.08–−29.40	>−29.40	
Case/Control	157/181	177/161	172/164	
Model 1 ^a^	1	1.26 (0.94–1.70)	1.21 (0.89–1.65)	1.00 (0.99–1.02)
Model 2 ^b^	1	0.95 (0.53–1.68)	1.46 (0.76–2.79)	1.01 (0.98–1.05)
HBS of CDQI	<20	20–30	>30	
Case/Control	156/227	144/157	206/122	
Model 1 ^a^	1	1.35 (0.99–1.84)	**2.56 (1.86–3.52)**	**1.03 (1.02–1.05)**
Model 2 ^b^	1	0.86 (0.52–1.44)	**2.58 (1.52–4.40)**	**1.03 (1.01–1.05)**
DQD of CDQI	<44.71	44.71–56.76	>56.76	
Case/Control	140/198	163/174	203/134	
Model 1 ^a^	1	1.32 (0.97–1.79)	**2.10 (1.54–2.86)**	**1.03 (1.02–1.04)**
Model 2 ^b^	1	0.76 (0.46–1.27)	**1.93 (1.15–3.24)**	**1.03 (1.01–1.05)**
LBS of CDBI	<−18	−18–−26	>−26	
Case/Control	113/241	181/154	212/111	
Model 1 ^a^	1	**2.56 (1.83–3.59)**	**3.97 (2.82–5.58)**	**1.06 (1.04–1.07)**
Model 2 ^b^	1	**3.68 (2.12–6.36)**	**5.75 (3.15–10.49)**	**1.08 (1.06–1.12)**
HBS of CDBI	<9	9–13	>13	
Case/Control	135/243	158/155	213/108	
Model 1 ^a^	1	**1.72 (1.27–2.33)**	**3.55 (2.55–4.96)**	**1.12 (1.09–1.15)**
Model 2 ^b^	1	**3.09 (1.82–5.24)**	**5.38 (2.97–9.75)**	**1.14 (1.09–1.20)**
DQD of CDBI	<28	28–38	>38	
Case/Control	114/245	156/166	236/95	
Model 1 ^a^	1	**1.98 (1.43–2.75)**	**5.19 (3.64–7.41)**	**1.06 (1.04–1.07)**
Model 2 ^b^	1	**2.98 (1.72–5.16)**	**7.94 (4.27–14.75)**	**1.08 (1.06–1.11)**
DII	88/250	199/138	219/118	
Case/Control	<−1.48	−1.48–0.80	>0.80	
Model 1 ^a^	1	**3.99 (2.80–5.67)**	**5.45 (3.75–7.90)**	**1.37 (1.28–1.47)**
Model 2 ^b^	1	**12.62 (6.09–26.16)**	**31.03 (12.33–78.09)**	**2.20 (1.81–2.68)**

Note: T1, T2, and T3 represent the tertiles of the Chinese dietary indices. ^c^ Continuous represents the result of each one-point changed in dietary index. Bold results indicate significance. Abbreviation: CDQI, Chinese Dietary Quality Index; CDBI, Chinese Dietary Balance Index; CHEI, Chinese Healthy Eating Index; DAI, Dietary Antioxidant Index; DII, Dietary Inflammation Index; DQD, dietary quality distance; HBS, high bound score; LBS, low bound score. ^a^ Model 1: unadjusted model. ^b^ Model 2: adjusted for age, BMI, occupation, education level, household income, high-risk residential areas, smoking status, history of allergies, history of head trauma, family history of cancer, physical activity, and energy intake.

**Table 3:** Adjusted Ors and 95% CI for the association between Chinese dietary indices and glioma of different pathological classifications.

Pathological Classification ^c^	Model 1 ^a^	*p*	Model 2 ^b^	*p*
Astrocytoma (*n* = 104)				
CHEI	**0.93 (0.90–0.96)**	**<0.001**	**0.89 (0.83–0.96)**	**0.001**
DAI	**0.93 (0.87–0.99)**	**0.016**	**0.01 (0.001–0.60)**	**0.027**
LBS of CDQI	0.99 (0.96–1.02)	0.659	1.01 (0.93–1.11)	0.798
HBS of CDQI	**1.03 (1.01–1.05)**	**0.020**	1.03 (0.99–1.08)	0.167
DQD of CDQI	**1.02 (1.00–1.05)**	**0.050**	1.04 (0.99–1.09)	0.131
LBS of CDBI	**1.06 (1.02–1.09)**	**0.001**	**1.16 (1.06–1.27)**	**0.001**
HBS of CDBI	**1.16 (1.08–1.24)**	**<0.001**	**1.19 (1.05–1.34)**	**0.005**
DQD of CDBI	**1.06 (1.03–1.10)**	**<0.001**	**1.17 (1.07–1.29)**	**0.001**
DII	**1.40 (1.19–1.63)**	**<0.001**	**5.49 (1.92–15.69)**	**0.001**
Glioblastoma (*n* = 237)				
CHEI	**0.93 (0.91–0.95)**	**<0.001**	**0.83 (0.77–0.90)**	**<0.001**
DAI	**0.94 (0.90–0.99)**	**0.007**	**0.71 (0.59–0.87)**	**0.001**
LBS of CDQI	1.00 (0.98–1.03)	0.731	1.02 (0.96–1.09)	0.554
HBS of CDQI	**1.04 (1.02–1.06)**	**<0.001**	1.02 (0.98–1.05)	0.411
DQD of CDQI	**1.04 (1.02–1.05)**	**<0.001**	1.02 (0.99–1.06)	0.244
LBS of CDBI	**1.05 (1.03–1.07)**	**<0.001**	**1.14 (1.06–1.22)**	**0.001**
HBS of CDBI	**1.11 (1.07–1.16)**	**<0.001**	**1.12 (1.04–1.22)**	**0.004**
DQD of CDBI	**1.05 (1.03–1.07)**	**<0.001**	**1.10 (1.05–1.15)**	**<0.001**
DII	**1.41 (1.26–1.56)**	**<0.001**	**2.21 (1.52–3.20)**	**<0.001**

Note: Due to the small sample size of oligodendroglioma (*n* = 67), no further analysis was conducted. These results represent the result of each one-point changed in dietary index. Bold results indicate significance. Abbreviation: CDQI, Chinese Dietary Quality Index; CDBI, Chinese Dietary Balance Index; CHEI, Chinese Healthy Eating Index; DAI, Dietary Antioxidant Index; DII, Dietary Inflammation Index; DQD, dietary quality distance; HBS, high bound score; LBS, low bound score. ^a^ Model 1: unadjusted model. ^b^ Model 2: adjusted for age, BMI, occupation, education level, household income, high-risk residential areas, smoking status, history of allergies, history of head trauma, family history of cancer, physical activity, and energy intake. ^c^ Dietary index per one-point increment.

**Table 4:** Adjusted Ors and 95% CI for the association between Chinese dietary indices and glioma of different grades.

Glioma Grading ^c^	Model 1 ^a^	*p*	Model 2 ^b^	*p*
Low grade (*n* = 105)				
CHEI	**0.93 (0.90–0.96)**	**<0.001**	**0.91 (0.86–0.96)**	**0.001**
DAI	**0.92 (0.87–0.99)**	**0.017**	**0.27 (0.12–0.61)**	**0.001**
LBS of CDQI	1.01 (0.97–1.04)	0.773	0.95 (0.85–1.05)	0.313
HBS of CDQI	**1.03 (1.01–1.06)**	**0.017**	1.03 (0.99–1.08)	0.164
DQD of CDQI	**1.03 (1.01–1.05)**	**0.014**	1.02 (0.98–1.07)	0.303
LBS of CDBI	**1.07 (1.03–1.10)**	**<0.001**	**1.12 (1.04–1.20)**	**0.002**
HBS of CDBI	**1.15 (1.06–1.24)**	**<0.001**	**1.29 (1.08–1.54)**	**0.005**
DQD of CDBI	**1.07 (1.04–1.10)**	**<0.001**	**1.13 (1.05–1.22)**	**0.001**
DII	**1.37 (1.17–1.59)**	**<0.001**	**3.21 (1.58–6.52)**	**0.001**
High grade (*n* = 328)				
CHEI	**0.93 (0.91–0.95)**	**<0.001**	**0.87 (0.83–0.91)**	**<0.001**
DAI	**0.95 (0.92–0.99)**	**0.005**	**0.69 (0.59–0.81)**	**<0.001**
LBS of CDQI	0.99 (0.98–1.02)	0.829	1.02 (0.98–1.08)	0.347
HBS of CDQI	**1.04 (1.02–1.06)**	**<0.001**	1.03 (0.99–1.06)	0.095
DQD of CDQI	**1.03 (1.02–1.05)**	**<0.001**	**1.03 (1.01–1.06)**	**0.035**
LBS of CDBI	**1.04 (1.03–1.06)**	**<0.001**	**1.10 (1.05–1.15)**	**<0.001**
HBS of CDBI	**1.11 (1.07–1.15)**	**<0.001**	**1.14 (1.07–1.22)**	**<0.001**
DQD of CDBI	**1.05 (1.03–1.07)**	**<0.001**	**1.09 (1.05–1.13)**	**<0.001**
DII	**1.36 (1.25–1.49)**	**<0.001**	**2.20 (1.67–2.89)**	**<0.001**

Note: These results represent the result of each one-point changed in dietary index. Bold results indicate significance. Abbreviation: CDQI, Chinese Dietary Quality Index; CDBI, Chinese Dietary Balance Index; CHEI, Chinese Healthy Eating Index; DAI, Dietary Antioxidant Index; DII, Dietary Inflammation Index; DQD, dietary quality distance; HBS, high bound score; LBS, low bound score. ^a^ Model 1: unadjusted model. ^b^ Model 2: adjusted for age, BMI, occupation, education level, household income, high-risk residential areas, smoking status, history of allergies, history of head trauma, family history of cancer, physical activity, and energy intake. ^c^ Dietary index per one-point increment.

## Data Availability

The data presented in this study are available on request from the corresponding author.

## Supplementary Materials

The following supporting information can be downloaded at: https://www.mdpi.com/article/10.3390/nu15163602/s1, Supplementary Material A: Supplemental methods: Components and scoring of Chinese dietary indices; Supplementary Material B: Table S1. Food items in the food frequency questionnaire. Table S2. The intake of major food groups in China in case group, control group, and national representative population in China. Table S3. Correlations between Chinese dietary indices among the case–control participants. Table S4. Sensitivity analysis of Chinese dietary indices and glioma. Table S5. Chinese dietary indices influenced the proportion of gliomas mediated by BMI. Figure S1. The flow chart of the case–control population matching. Refs [26,27,28,30,61,64,86,87,88] are cited in the Supplementary Materials.
