# Supplementary material for: Chinese Dietary Indices and Glioma: New Insights of a Case–Control Study in the Chinese Population
Source: Nutrients. 2023 Aug 17;15(16):3602. doi: 10.3390/nu15163602 (PMC10457799; doi:10.3390/nu15163602)
Supplement: Supplementary file 1 [file nutrients-15-03602-s001.zip › Supplementary Material A_Revision2.pdf]

Supplementary Material A

Supplemental methods: Components and scoring of Chinese dietary indices

China Healthy Eating Index

The China Healthy Eating Index (CHEI) is a dietary index developed based on the Chinese Dietary Guidelines and has been applied to the China Health and Nutrition Survey to assess the consistency of the dietary situation with the Chinese Dietary Guidelines [1]. The evaluation of the index involved 17 components. On the one hand, they were adequacy components, including total grains, whole grains and mixed beans, tubers, total vegetables, dark vegetables, fruits, dairy, soybeans, fish and seafood, poultry, eggs, seeds, and nuts. On the other hand, there were limitation components, including red meat, cooking oil, sodium, added sugars, and alcohol. All components were scored based on energy density (intake per 1000 calories). The score of the adequacy component increased with the increase in intake, while the score of the limitation component was the opposite. Fruits, cooking oils, and sodium have a maximum score of 10, and other food groups have a maximum score of 5, so their overall score ranged from 0-100.

Chinese Dietary Quality Index

The Chinese Dietary Quality Index (CDQI) was formulated based on the American Dietary Quality Index and the characteristics of the Chinese diet and was used to evaluate the quality of individual diets and reflect the nutritional problems in the dietary structure of Chinese residents [2]. The index included a total of 10 components: diet variety (-12-0), fruits and vegetables (-12-0), protein (10-0), calcium (-10-0), saturated fatty acids (0-10), sodium (0-10), alcohol (0-6), energy (-10-10), total carbohydrate (-10-10) and total fat (-10-10). According to the actual situation of each component, a two-way score was set, with negative scores reflecting insufficient intake and positive scores reflecting excessive intake, and the low bound score (LBS, sum of negative points), high bound score (HBS, sum of positive points) and dietary quality distance (DQD, the sum of absolute values of each component's score, reflecting nutritional imbalance) were calculated separately.

Chinese Dietary Balance Index

The Chinese Dietary Balance Index (CDBI) was an indicator used to evaluate the dietary balance of individuals based on the dietary structure of Chinese residents [3,4]. The index consisted of 14 food components in 8 food groups, including cereals (-12-12), vegetables (-6-0), fruits (-6-0), dairy (-6-0), soybeans (-6-0), red meat, poultry meat and their products (-4-4), fish and seafood (-4-0), eggs (-4-4), cooking oils (0-6), alcohol (0-6), added sugars (0-6), salt (0-6), diet variety (-12-0) and drinking water (-12-0). Unlike other indices, CDBI gave different recommended amounts of food based on different energy levels. When each component reached the recommended amount, the score was 0. Negative scores reflected insufficient intake and positive scores reflected excessive intake, and LBS (sum of negative points), HBS (sum of positive points), and DQD (sum of absolute values of each component's score, reflecting nutritional imbalance) were calculated separately.

Dietary antioxidant index

The dietary antioxidant index (DAI) was proposed by Wright et al. to measure the overall antioxidant capacity of the diet [5]. The index was made up of six vitamins and minerals with antioxidant capacity, including manganese, zinc, selenium, vitamin A, vitamin C, and vitamin E. When calculating, it needed to be standardized first. Briefly, the intake of each antioxidant

nutrient subtracted the mean and divided by the standard deviation, and finally, the scores were added up to the DAI [6].

Dietary inflammation index

The dietary Inflammation Index (DII) was a quantitative indicator used to assess the overall impact of diet on inflammation in the body. We applied the method of Shivappa et al. to normalize foods or nutrients with potential pro- or anti-inflammatory effects (intake subtracted the standard mean and divided this value by its standard deviation) and converted this value to percentile scores [7]. To achieve a symmetric distribution, the values were centered on 0 and ranged between −1 (maximum anti-inflammatory effect) and +1 (maximum pro-inflammatory effect), with each percentile score doubled and then subtracted "1". Finally, they multiplied by their respective "Overall inflammatory effect score" and added up the scores to form the total score of the individual's DII. Since some foods were not common in the Chinese diet, this DII calculation included 28 food parameters.

References:

1. Yuan, Y.Q.; Li, F.; Dong, R.H.; Chen, J.S.; He, G.S.; Li, S.G.; Chen, B. The Development of a Chinese Healthy Eating Index and Its Application in the General Population. *Nutrients* **2017**, 9, doi:10.3390/nu9090977.
2. Stookey, J.D.; Wang, Y.; Ge, K.; Lin, H.; Popkin, B.M. Measuring diet quality in china: the INFH-UNC-CH diet quality index. *Eur J Clin Nutr* **2000**, 54, 811-821, doi:10.1038/sj.ejcn.1601098.
3. Zou, Z.; Li, Q.; Huang, H.; Liu, D.; Tan, R.; Xu, S. Unfavorable dietary quality and overweight or obesity in kidney transplant recipients as judged by the Chinese diet balance index 2016 (DBI-16). *Asia Pac J Clin Nutr* **2022**, 31, 551-560, doi:10.6133/apjcn.202209_31(3).0023.
4. He, Y.N.; F, Y.H; X,J. Update of the Chinese diet balance index: DBI_16. *Acta Nutrimenta Sinica* **2018**, 40, 526-530.
5. Wright, M.E.; Mayne, S.T.; Stolzenberg-Solomon, R.Z.; Li, Z.; Pietinen, P.; Taylor, P.R.; Virtamo, J.; Albanes, D. Development of a comprehensive dietary antioxidant index and application to lung cancer risk in a cohort of male smokers. *Am J Epidemiol* **2004**, 160, 68-76, doi:10.1093/aje/kwh173.
6. Vahid, F.; Rahmani, D.; Davoodi, S.H. Validation of Dietary Antioxidant Index (DAI) and investigating the relationship between DAI and the odds of gastric cancer. *Nutr Metab* **2020**, 17, 102, doi:10.1186/s12986-020-00529-w.
7. Shivappa, N.; Steck, S.E.; Hurley, T.G.; Hussey, J.R.; Hebert, J.R. Designing and developing a literature-derived, population-based dietary inflammatory index. *Public Health Nutr* **2014**, 17, 1689-1696, doi:10.1017/S1368980013002115.
