# Supplementary material for: Chinese Dietary Indices and Glioma: New Insights of a Case–Control Study in the Chinese Population
Source: Nutrients. 2023 Aug 17;15(16):3602. doi: 10.3390/nu15163602 (PMC10457799; doi:10.3390/nu15163602)
Supplement: Supplementary file 1 [file nutrients-15-03602-s001.zip › Supplementary Material B_Revision2.pdf]

Supplementary Material

Table S1. Food items in the food frequency questionnaire.

Table S2. The intake of major food groups in China in case group, control group, and national representative population in China.

Table S3. Correlations between Chinese dietary indices among the case-control participants.

Table S4. Sensitivity analysis of Chinese dietary indices and glioma.

Table S5. Chinese dietary indices influenced the proportion of gliomas mediated by BMI.

Figure S1. The flow chart of the case control population matching.

Table S1. Food items in the food frequency questionnaire.

Food group (n)	Items
Refined grains (9)	Rice, steamed buns, scones, porridge, deep-fried dough sticks, noodles, instant noodles, vermicelli, dumplings
Whole grains (2)	Corn， millet and oats
Tubers (2)	Potatoes, sweet potatoes
Legume and Products (5)	Soy, soy milk, tofu, other soy products, other legumes
Vegetables (23)	Green leafy vegetables, kale, cabbage, celery, eggplant, cucumber, white gourd, pumpkin, carrot, radish, green shoot, broccoli, bell pepper, pepper, tomato, lotus root, chive, garlic moss, bitter melon, onion, green bean, zucchini, cauliflower
Fungi and algae (4)	Mushrooms, Auricularia auricula, tremella, kelp
Fruits (20)	Apple, pear, orange, banana, peach, watermelon, melon, grape, pineapple, kiwi fruit, strawberries, cherries, blueberries, mulberries, shaddock, hawthorn, pitaya, Chinese date, mango, cherry tomato
Red meat (4)	Pork (muscle), pork (fat & muscle), beef, mutton
Poultry (2)	Chicken, duck
Animal offal (4)	Liver, blood, intestine, other viscera
Fish and seafood (5)	Freshwater fish, sea fish, shrimp, crab, mollusk
Egg (1)	Fresh egg
Dairy products (4)	Milk, yogurt, milk powder, cheese
Nuts (4)	Peanuts, sunflower seeds, walnuts, other nuts
Sweet food (5)	Bread, honey, biscuit, cake, Chinese pastry
Sugary drink (3)	Carbonated drinks, fruit and vegetable drinks, functional drinks
Tea and coffee (3)	Green tea, black tea, coffee
Condiment (4)	Salt, animal oil, olive oil, other vegetable oil
Curing food (3)	Sauerkraut, pickle, fermented bean curd
Processed products (3)	Bacon, sausage and other smoked meat, salted duck eggs, preserved eggs
Alcohol (4)	Beer, spirit, wine, rice wine

Table S2. The intake of major food groups in China in case group, control group, and national representative population in China.

Food group	Case	Control	Representative data 1 [a]	Representative data 2 [b]
Grains and products(g/d)	231.61±106.64	193.32±105.31	320.5	263.9
Tubers(g/d)	28.20±43.42	46.43±58.95	35.8	35.5
Legume and products(g/d)	8.78±10.43	15.02±19.63	15.2	15.6
Vegetables(g/d)	219.08±155.89	409.05±245.67	269.4	286.5
Fruits(g/d)	167.10±152.56	249.70±191.21	40.7	55.7
Red meat and products(g/d)	65.85±55.95	65.18±52.75	82.5	-
Animal offal	2.64±13.10	4.32±8.68	2.5	-
Poultry(g/d)	14.76±18.94	14.45±19.89	14.7	-
Red meat and poultry(g/d)	80.61±64.58	79.63±61.16	-	98.0
Egg(g/d)	51.10±34.96	48.54±34.79	24.3	30.4
Fish and seafood (g/d)	14.04±22.56	24.45±33.66	23.7	29.7
Nuts(g/d)	10.85±21.17	9.95±15.33	3.81	4.7

a. Representative data 1 sourced from the 2015 Report on Nutrition and Chronic Disease Status of Chinese Residents [1].
b. Representative data 2 sourced from Chinese Center for Disease Control and Prevention [2].

References

1. Gu, J.F. Interpretation of the Report on Nutrition and Chronic Disease Status of Chinese Residents (2015). *Acta Nutrimenta Sinica*. **2016**, 38, 525-529., doi: 10.13325/j.cnki.acta.nutr.sin.2016.06.004.
2. Chinese Center for Disease Control and Prevention. https://www.chinanutri.cn/sjnj/swsryssjg/zgjmswsrl/zgjmglswpjsrl/201512/t20151230_123992.html (Accessed on July 24, 2023).

Table S3. Correlations between Chinese dietary indices among the case-control participants.

	CHEI	LBS of CDQI	HBS of CDQI	DQD of CDQI	LBS of CDBI	HBS of CDBI	DQD of CDBI	DAI	DII
CHEI	1.000	0.306*	-0.122*	-0.312*	0.793*	-0.446*	-0.821*	0.401*	-0.675*
LBS of CDQI	0.306*	1.000	0.250*	-0.418*	0.350*	-0.159*	-0.364*	0.706*	-0.532*
HBS of CDQI	-0.122*	0.250*	1.000	0.737*	0.050	0.227*	0.048	0.156*	0.003
DQD of CDQI	-0.312*	-0.418*	0.737*	1.000	-0.189*	0.298*	0.284*	-0.331*	0.362*
LBS of CDBI	0.793*	0.350*	0.050	-0.189*	1.000	-0.271*	-0.906*	0.410*	-0.617*
HBS of CDBI	-0.446*	-0.159*	0.227*	0.298*	-0.271*	1.000	0.627*	-0.174*	0.317*
DQD of CDBI	-0.821*	-0.364*	0.048	0.284*	-0.906*	0.627*	1.000	-0.419*	0.636*
DAI	0.401*	0.706*	0.156*	-0.331*	0.410*	-0.174*	-0.419*	1.000	-0.861*
DII	-0.675*	-0.532*	0.003	0.362*	-0.617*	0.317*	0.636*	-0.861*	1.000

*P<0.001.

Table S4. Sensitivity analysis of Chinese dietary indices and glioma.

Group [a]	Model 1 [b]	*P*	Model 2 [c]	*P*
**Age**				
**≤40(n=500)**				
CHEI	**0.95(0.93-0.97)**	**<0.001**	**0.94(0.92-0.96)**	**<0.001**
DAI	0.97(0.93-1.01)	0.117	**0.63(0.55-0.72)**	**<0.001**
LBS of CDQI	0.99(0.97-1.02)	0.577	1.01(0.97-1.04)	0.682
HBS of CDQI	**1.03(1.01-1.05)**	**0.001**	1.02(0.99-1.04)	0.114
DQD of CDQI	**1.02(1.01-1.04)**	**0.005**	1.02(0.99-1.04)	0.077
LBS of CDBI	**1.04(1.02-1.06)**	**< 0.001**	**1.05(1.03-1.08)**	**< 0.001**
HBS of CDBI	**1.10(1.06-1.14)**	**<0.001**	**1.08(1.03-1.13)**	**0.001**
DQD of CDBI	**1.04(1.03-1.06)**	**<0.001**	**1.05(1.03-1.08)**	**<0.001**
DII	**1.25(1.14-1.37)**	**<0.001**	**1.91(1.60-2.26)**	**<0.001**
**>41(n=512)**				
CHEI	**0.91(0.89-0.93)**	**<0.001**	**0.89(0.86-0.91)**	**<0.001**
DAI	**0.91(0.87-0.95)**	**<0.001**	**0.65(0.58-0.73)**	**<0.001**
LBS of CDQI	1.01(0.99-1.03)	0.326	1.01(0.98-1.05)	0.575
HBS of CDQI	**1.03(1.02-1.05)**	**<0.001**	**1.04(1.02-1.06)**	**<0.001**
DQD of CDQI	**1.04(1.02-1.05)**	**<0.001**	**1.04(1.02-1.06)**	**<0.001**
LBS of CDBI	**1.07(1.05-1.10)**	**< 0.001**	**1.09(1.06-1.12)**	**< 0.001**
HBS of CDBI	**1.13(1.08-1.17)**	**<0.001**	**1.09(1.05-1.14)**	**<0.001**
DQD of CDBI	**1.07(1.05-1.09)**	**<0.001**	**1.08(1.05-1.10)**	**<0.001**
DII	**1.51(1.38-1.66)**	**<0.001**	**2.12(1.80-2.49)**	**<0.001**
**Sex**				
**Male(n=568)**				
CHEI	**0.94(0.92-0.95)**	**<0.001**	**0.92(0.90-0.94)**	**<0.001**
DAI	**0.96(0.92-0.99)**	**0.010**	**0.63(0.56-0.70)**	**<0.001**
LBS of CDQI	0.99(0.97-1.01)	0.203	1.01(0.98-1.04)	0.581
HBS of CDQI	**1.03(1.02-1.05)**	**<0.001**	**1.02(1.01-1.04)**	**0.013**
DQD of CDQI	**1.02(1.01-1.04)**	**0.004**	**1.02(1.01-1.04)**	**0.007**
LBS of CDBI	**1.04(1.02-1.05)**	**< 0.001**	**1.06(1.04-1.08)**	**< 0.001**
HBS of CDBI	**1.10(1.06-1.14)**	**<0.001**	**1.09(1.05-1.14)**	**<0.001**
DQD of CDBI	**1.04(1.03-1.06)**	**<0.001**	**1.06(1.04-1.08)**	**<0.001**
DII	**1.28(1.19-1.39)**	**<0.001**	**1.90(1.65-2.19)**	**<0.001**
**Female(n=444)**				
CHEI	**0.90(0.88-0.93)**	**<0.001**	**0.90(0.87-0.93)**	**<0.001**
DAI	**0.91(0.87-0.96)**	**<0.001**	**0.68(0.59-0.78)**	**<0.001**
LBS of CDQI	**1.03(1.00-1.05)**	**0.038**	1.00(0.96-1.05)	0.880
HBS of CDQI	**1.03(1.02-1.05)**	**<0.001**	**1.03(1.01-1.06)**	**0.004**
DQD of CDQI	**1.04(1.02-1.06)**	**<0.001**	**1.03(1.01-1.06)**	**0.004**
LBS of CDBI	**1.10(1.07-1.13)**	**< 0.001**	**1.10(1.06-1.14)**	**< 0.001**
HBS of CDBI	**1.13(1.08-1.17)**	**<0.001**	**1.09(1.03-1.14)**	**0.002**
DQD of CDBI	**1.09(1.06-1.11)**	**<0.001**	**1.09(1.06-1.12)**	**<0.001**
DII	**1.50(1.35-1.66)**	**<0.001**	**2.24(1.80-2.77)**	**<0.001**

**BMI**				
**≤23.31(n=506)**				
CHEI	**0.94(0.92-0.95)**	**<0.001**	**0.93(0.91-0.95)**	**<0.001**
DAI	**0.93(0.90-0.97)**	**0.001**	**0.70(0.61-0.80)**	**<0.001**
LBS of CDQI	**1.02(0.99-1.04)**	**0.100**	1.01(0.98-1.05)	0.525
HBS of CDQI	**1.03(1.01-1.04)**	**0.001**	**1.03(1.01-1.05)**	**0.013**
DQD of CDQI	**1.03(1.02-1.05)**	**<0.001**	**1.03(1.01-1.05)**	**0.005**
LBS of CDBI	**1.04(1.02-1.06)**	**< 0.001**	**1.05(1.03-1.08)**	**< 0.001**
HBS of CDBI	**1.12(1.08-1.17)**	**<0.001**	**1.09(1.04-1.14)**	**<0.001**
DQD of CDBI	**1.05(1.04-1.07)**	**<0.001**	**1.05(1.03-1.08)**	**<0.001**
DII	**1.32(1.21-1.44)**	**<0.001**	**1.79(1.52-2.11)**	**<0.001**
**>23.31(n=506)**				
CHEI	**0.92(0.90-0.94)**	**<0.001**	**0.91(0.88-0.93)**	**<0.001**
DAI	**0.93(0.90-0.97)**	**0.001**	**0.61(0.54-0.69)**	**<0.001**
LBS of CDQI	0.99(0.97-1.01)	0.336	1.00(0.96-1.03)	0.907
HBS of CDQI	**1.03(1.02-1.05)**	**<0.001**	**1.03(1.01-1.05)**	**0.004**
DQD of CDQI	**1.03(1.01-1.04)**	**0.001**	**1.03(1.01-1.05)**	**0.005**
LBS of CDBI	**1.07(1.05-1.09)**	**< 0.001**	**1.08(1.06-1.11)**	**< 0.001**
HBS of CDBI	**1.10(1.06-1.14)**	**<0.001**	**1.09(1.05-1.14)**	**<0.001**
DQD of CDBI	**1.06(1.04-1.08)**	**<0.001**	**1.07(1.05-1.10)**	**<0.001**
DII	**1.44(1.31-1.59)**	**<0.001**	**2.21(1.86-2.62)**	**<0.001**
**Education level**				
**University and above(n=627)**				
CHEI	**0.94(0.92-0.95)**	**<0.001**	**0.93(0.91-0.94)**	**<0.001**
DAI	**0.96(0.92-0.99)**	**0.014**	**0.67(0.60-0.74)**	**<0.001**
LBS of CDQI	0.99(0.97-1.01)	0.389	1.00(0.97-1.03)	0.944
HBS of CDQI	**1.04(1.02-1.05)**	**<0.001**	**1.03(1.01-1.05)**	**0.001**
DQD of CDQI	**1.03(1.01-1.04)**	**<0.001**	**1.03(1.01-1.05)**	**0.001**
LBS of CDBI	**1.04(1.02-1.06)**	**< 0.001**	**1.06(1.03-1.08)**	**< 0.001**
HBS of CDBI	**1.11(1.07-1.15)**	**<0.001**	**1.09(1.05-1.13)**	**<0.001**
DQD of CDBI	**1.05(1.03-1.06)**	**<0.001**	**1.06(1.04-1.08)**	**<0.001**
DII	**1.30(1.20-1.41)**	**<0.001**	**1.89(1.64-2.18)**	**<0.001**
**Household income**				
**>3,000 ¥/month(n=871)**				
CHEI	**0.92(0.91-0.94)**	**<0.001**	**0.92(0.90-0.93)**	**<0.001**
DAI	**0.93(0.90-0.96)**	**<0.001**	**0.65(0.59-0.71)**	**<0.001**
LBS of CDQI	1.01(0.99-1.03)	0.265	1.01(0.99-1.04)	0.339
HBS of CDQI	**1.03(1.02-1.04)**	**<0.001**	**1.03(1.02-1.05)**	**<0.001**
DQD of CDQI	**1.03(1.02-1.04)**	**<0.001**	**1.03(1.02-1.05)**	**<0.001**
LBS of CDBI	**1.07(1.05-1.09)**	**< 0.001**	**1.07(1.05-1.10)**	**< 0.001**
HBS of CDBI	**1.12(1.09-1.15)**	**<0.001**	**1.10(1.06-1.14)**	**<0.001**

DQD of CDBI	**1.07(1.05-1.08)**	**<0.001**	**1.07(1.05-1.09)**	**<0.001**
DII	**1.42(1.32-1.52)**	**<0.001**	**1.97(1.74-2.22)**	**<0.001**
**Smoking status**				
**Never smoking(n=735)**				
CHEI	**0.93(0.92-0.95)**	**< 0.001**	**0.93(0.92-0.95)**	**< 0.001**
DAI	**0.94(0.90-0.97)**	**< 0.001**	**0.69(0.63-0.77)**	**< 0.001**
LBS of CDQI	1.01(0.99-1.03)	0.399	1.00(0.97-1.03)	0.993
HBS of CDQI	**1.03(1.02-1.05)**	**< 0.001**	**1.03(1.01-1.05)**	**< 0.001**
DQD of CDQI	**1.03(1.02-1.05)**	**< 0.001**	**1.03(1.01-1.04)**	**0.001**
LBS of CDBI	**1.05(1.03-1.07)**	**< 0.001**	**1.05(1.03-1.07)**	**< 0.001**
HBS of CDBI	**1.13(1.09-1.16)**	**< 0.001**	**1.09(1.05-1.13)**	**< 0.001**
DQD of CDBI	**1.06(1.04-1.07)**	**< 0.001**	**1.05(1.03-1.07)**	**< 0.001**
DII	**1.35(1.26-1.46)**	**< 0.001**	**1.82(1.60-2.08)**	**< 0.001**
**History of allergies**				
**No(n=899)**				
**CHEI**	**0.93(0.92-0.94)**	**<0.001**	**0.92(0.90-0.93)**	**<0.001**
DAI	**0.93(0.90-0.96)**	**<0.001**	**0.64(0.58-0.70)**	**<0.001**
LBS of CDQI	1.00(0.99-1.02)	0.637	1.01(0.98-1.04)	0.471
HBS of CDQI	**1.03(1.02-1.04)**	**<0.001**	**1.02(1.01-1.04)**	**0.001**
DQD of CDQI	**1.03(1.02-1.04)**	**<0.001**	**1.03(1.01-1.04)**	**<0.001**
LBS of CDBI	**1.06(1.04-1.08)**	**< 0.001**	**1.08(1.06-1.10)**	**< 0.001**
HBS of CDBI	**1.12(1.08-1.15)**	**<0.001**	**1.09(1.06-1.13)**	**<0.001**
DQD of CDBI	**1.06(1.05-1.07)**	**<0.001**	**1.07(1.05-1.09)**	**<0.001**
DII	**1.38(1.29-1.47)**	**<0.001**	**1.95(1.73-2.20)**	**<0.001**
**Family history of cancer**				
**No(n=753)**				
CHEI	**0.93(0.92-0.94)**	**<0.001**	**0.92(0.91-0.94)**	**<0.001**
DAI	**0.93(0.90-0.96)**	**<0.001**	**0.64(0.58-0.71)**	**<0.001**
LBS of CDQI	1.00(0.99-1.02)	0.719	1.01(0.98-1.04)	0.600
HBS of CDQI	**1.03(1.02-1.05)**	**<0.001**	**1.02(1.01-1.04)**	**0.005**
DQD of CDQI	**1.03(1.02-1.05)**	**<0.001**	**1.02(1.01-1.04)**	**0.002**
LBS of CDBI	**1.05(1.04-1.07)**	**< 0.001**	**1.07(1.05-1.09)**	**< 0.001**
HBS of CDBI	**1.13(1.09-1.17)**	**<0.001**	**1.11(1.07-1.15)**	**<0.001**
DQD of CDBI	**1.06(1.05-1.07)**	**<0.001**	**1.07(1.05-1.09)**	**<0.001**
DII	**1.38(1.28-1.48)**	**<0.001**	**1.99(1.74-2.27)**	**<0.001**

Note: These results represented the result of each 1-point changed in dietary index. Bold results indicate significance. Abbreviation: CDQI, Chinese Dietary Quality Index; CDBI, Chinese Dietary Balance Index; CHEI, Chinese Healthy Eating Index; DAI, Dietary antioxidant index; DII, Dietary inflammation index; DQD, dietary quality distance; HBS, high bound score; LBS. low bound score.

[a]. Unconditional logistic regression model was used for sensitivity analysis.

[b] Model 1: Unadjusted model.

[c] Model 2: Adjusted for age, BMI, occupation, education level, household income, high-risk residential areas, smoking status, history of allergies, history of head trauma, family history of cancer, physical activity, and energy intake (except for corresponding hierarchical variables).

Table S5. Chinese dietary indices influenced the proportion of gliomas mediated by BMI.

Dietary index[a]	Total effect	ADE	ACME	Proportion mediated (%)
CHEI	-0.16(-0.17, -0.14)	-0.15(-0.16, -0.14)	-0.003(-0.007, 0.00)	2.18
P	<0.001	<0.001	0.010	0.010
LBS of CDQI	-0.05(-0.09, -0.01)	-0.05(-0.09, 0)	-0.003(-0.008, 0)	5.27
P	0.024	0.036	0.354	0.366
HBS of CDQI	0.08(0.05, 0.11)	0.08(0.05, 0.11)	0.002(-0.001, 0.01)	2.68
P	<0.001	<0.001	0.300	0.300
DQD of CDQI	0.07(0.04, 0.10)	0.07(0.04, 0.10)	0.002(-0.001, 0.01)	3.17
P	<0.001	<0.001	0.210	0.210
LBS of CDBI	-0.13(-0.15, -0.10)	-0.13(-0.15, -0.10)	-0.003(-0.008, 0)	2.54
P	<0.001	<0.001	0.074	0.074
HBS of CDBI	0.11(0.08, 0.13)	0.11(0.08, 0.13)	0.003(-0.001, 0.01)	2.63
P	<0.001	<0.001	0.120	0.120
DQD of CDBI	0.15(0.12, 0.16)	0.14(0.12, 0.16)	0.003(-0.002, 0.01)	1.99
P	<0.001	<0.001	0.066	0.066
DAI	-0.15(-0.16, -0.12)	-0.15(-0.16, -0.12)	-0.001(-0.006, 0)	0.94
P	<0.001	<0.001	0.520	0.520
DII	0.16(0.15, 0.17)	0.16(0.15, 0.17)	0.003(-0.0001, 0.01)	1.69
P	<0.001	<0.001	0.056	0.056

Note: ACME, average causal mediation effects; ADE, average direct effects; CDQI, Chinese Dietary Quality Index; CDBI, Chinese Dietary Balance Index; CHEI, Chinese Healthy Eating Index; DAI, Dietary antioxidant index; DII, Dietary inflammation index; DQD, dietary quality distance; HBS, high bound score; LBS. low bound score.

a. Adjusted for age, occupation, education level, household income, high-risk residential areas, smoking status, history of allergies, history of head trauma, family history of cancer, physical activity, and energy intake.

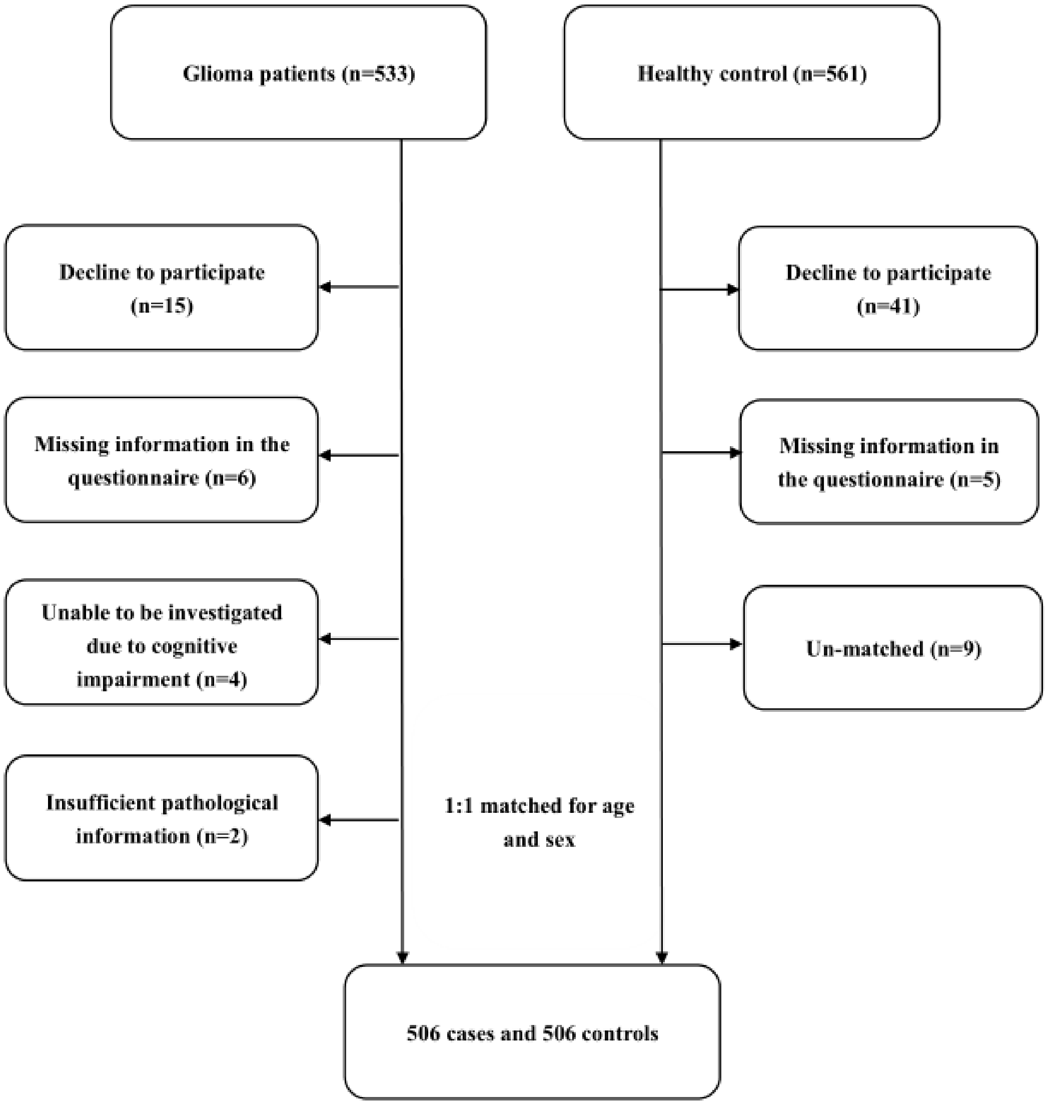

Figure S1. The flow chart of the case control population matching.
